# Trends in Adolescent Sexual and Reproductive Health Outcomes Before and Into the COVID-19 Pandemic in Burkina Faso and Kenya: Evidence From Panel Data

**DOI:** 10.1016/j.jadohealth.2024.04.023

**Published:** 2024-08

**Authors:** Claire Silberg, Caroline Moreau, Celia Karp, Fiacre Bazié, Peter Gichangi, Georges Guiella, Yentéma Onadja, Mary Thiongo, Philip Anglewicz

**Affiliations:** a Department of Population, Family and Reproductive Health, Bloomberg School of Public Health, Johns Hopkins University, Baltimore, Maryland; b Institut Supérieur des Sciences de la Population (ISSP), Université Joseph KI-Zerbo, Ouagadougou, Burkina Faso; c International Centre for Reproductive Health-Kenya, Nairobi, Kenya; d Technical University of Mombasa, Mombasa, Kenya

**Keywords:** Adolescents, COVID-19, Sexual and reproductive health, Family planning

## Abstract

**Purpose:**

Many predicted that COVID-19 would have a substantial impact on the sexual and reproductive health (SRH) trajectories of adolescents in sub-Saharan Africa. The lack of longitudinal data with information collected before and into the pandemic has limited investigation into this topic.

**Methods:**

We performed a secondary analysis using nationally representative longitudinal data from Kenya and Burkina Faso, collected at three time points (pre–COVID-19 in late 2019, and during COVID-19 in 2020 and 2021), to determine the extent to which SRH outcomes and behaviors, including pregnancy, contraceptive use, partnership status, and sexual activity, changed during the pandemic among adolescent women.

**Results:**

Among adolescents aged 15–19 years (Kenya n = 1,893, Burkina Faso n = 1,422), there was a reduction in both partnership and pregnancy in 2021 as compared to pre-COVID 2019. Contraception use significantly increased between 2019 and 2021 in Kenya only (adjusted odds ratio [aOR]: 1.42, 95% confidence interval [CI] 1.03–1.97). COVID-19–related household income loss was associated with a decline in sexual activity among unmarried Kenyan girls (aOR: 0.47, 95% CI 0.25–0.87) and lower odds of pregnancy in Burkina Faso (aOR: 0.13, 95% CI 0.02–0.91). We did not find a relationship between COVID-19 measures and initiation of partnership or marriage in either setting.

**Discussion:**

Contrary to expectations, our results suggest that COVID-19 did not have a consistent or sustaining impact on adolescent SRH and behaviors in Burkina Faso and Kenya. Further research is needed to assess the longer-term implications of the pandemic on adolescent social and health outcomes.

**Implications and Contribution**

While previous research has focused on the immediate impacts and consequences of the COVID-19 pandemic on adolescent health and wellbeing, this study used longitudinal data from shortly before COVID-19 and two time points into the pandemic (2020 and 2021) to examine changes in sexual and reproductive health outcomes among adolescents.

Many predicted that the COVID-19 pandemic would result in widespread and deleterious sexual and reproductive health (SRH) outcomes among adolescents, particularly in low-income contexts such as sub-Saharan Africa (SSA) [[1], [2], [3], [4], [5], [6]]. In particular, it was purported that the pandemic would exacerbate existing adolescent sexual and reproductive health (ASRH) inequities and further marginalize youth populations [1,4]. School and social disruptions were expected to accelerate time to key developmental milestones, including sexual initiation and early marriage, potentially resulting in increased childbearing and unintended births [7]. Projections estimated that up to 10 million girls globally were at risk of becoming child brides due to school closures (UNICEF, 2021) [8]. Furthermore, some predicted that disruptions to the medical supply chain and reductions in access to “nonessential” healthcare services would adversely impact contraceptive use and delivery of critical antenatal and maternity care services [6,9,10]. United Nations Population Fund projected 1.4 million unintended births globally as a result of disruptions to family planning services (UNFPA, 2021). If these conjectures and projections held true, the negative impacts of the COVID-19 pandemic would have resulted in unprecedented shifts in SRH outcomes for adolescents globally, potentially reversing decades of progress in improving the health and lives of this population.

Evidence from early in the pandemic suggests that some of these concerns were valid. Young men and women in Kenya, South Africa, and Uganda reported increased depression, isolation, and anxiety at the onset of the pandemic, and described disruptions to healthcare and social services [[11], [12], [13], [14]]. A mixed-methods study from urban Kenya found that adolescent girls faced difficulty in procuring contraceptives and menstrual hygiene products and experienced job loss, leading to a greater reliance on transactional relationships [12]. In Uganda, youth reported entering partnerships as a means of escape from the idleness of COVID-19 and the stressors of home life [14]. Some youth in urban Nairobi, Kenya, reported that the pandemic resulted in less time spent with partners, while others reported that school closures increased their time spent with partners [15]. Quantitative evidence from Western Kenya revealed that, compared to girls who consistently remained in school, girls who experienced school disruption were more likely to be sexually active and were less likely to report that their first sexual experience was desired [16].

Emerging research on the relationship between COVID-19 and contraception has not shown a long-term or detrimental impact of the pandemic in SSA. Evidence showed an initial decline in adolescent provision of family planning services between March and May of 2020, accompanied by a rebound in the following months (United Nations Population Fund, 2020) [17,18]. A longer-term analysis of contraceptive dynamics found that more women (aged 15–49 years) actually adopted a contraceptive method in the first 6 months of the pandemic than discontinued their method in both Kenya and Burkina Faso [19]. Population-level panel data from four countries in SSA found no significant change in contraceptive use among all married women aged 15–49 years [20]. Finally, there was no evidence of a significant spike in pregnancies during the first 6 months of the COVID-19 pandemic among Kenyan adolescents [21].

While informative, the body of research on the impact of COVID-19 on ASRH has important limitations. Much of the evidence comes from studies conducted early in the pandemic or immediately after COVID-19 mitigation lockdowns were lifted. Most research is also limited in geographic scope, relying on relatively small samples for qualitative research or nonrepresentative samples for quantitative studies. Robust evidence on the association between disrupted social dynamics, sexual behaviors, and sexual health outcomes remains scarce or nonexistent. While initial studies provide an important snapshot into the social conditions of youth populations during the early months of the pandemic, this research is inadequate for examining if COVID-19–related factors widely influenced SRH outcomes and led to longer-term trends [[11], [12], [13], [14]]. Few studies have the longitudinal data necessary to measure changes in behaviors from prepandemic conditions to COVID-19 conditions as well as the potential longer-term consequences on SRH and trajectories related to these economic and social changes.

Burkina Faso and Kenya are instructive settings in which to examine the impact of COVID-19 on ASRH outcomes, since they are characterized by different developmental, cultural, and ASRH circumstances. Burkina Faso has a young and rapidly growing population, with two of three individuals under the age of 25 years; children under 15 represent almost half of the total population [22]. Child marriage is still common practice, with more than half of all women aged 18–49 years having been child brides (UNICEF, 2021). The proportion of young women married as children has decreased by nearly 15% over the past decade (UNICEF, 2021) and the median age of marriage has increased to 17.8 years [23]. In Kenya, around 38% of the total population is aged less than 15 years. By 2014, the median age of marriage had risen to 19.5 years for women in rural regions and 21.5 years for women residing in urban areas [24]. Comparing pregnancy trends, 20% of Burkinabe adolescents and 15% of Kenyan adolescents aged 15–19 years have ever been pregnant [24]. In both settings, adolescent women with lower educational attainment or who reside in rural areas have higher rates of early pregnancy [23,25]. Among all women, the modern contraceptive prevalence rate was 46% in Kenya and 28% in Burkina Faso, and the most common contraceptive methods used were implants and injectables in both countries [26,27].

COVID-19 was identified in Burkina Faso and Kenya in March of 2020, prompting governments to respond with border closings, school closures, travel restrictions, banned social gatherings, and mandatory dawn-to-dusk curfews [11,28]. In Burkina Faso, many vulnerable and hard-to-reach populations were without support or guidance on COVID-19 prevention and transmission control [29]. The government lifted the national curfew on June 13, 2020, and public schools were reopened by July of 2020 [30]. However, ongoing civil conflict, population displacement, and insecurity kept more than 500,000 children from returning to school fulltime [31]. In Kenya, the Government's initial response focused on high transmission zones; namely, preventing movement into and out of Nairobi County, Mombasa County, and three other designated counties. Outpatient maternal facilities and government satellite clinics were either shutdown or converted into COVID-19 isolation units. The Government of Kenya began lifting social restrictions and lockdowns by July of 2020 and announced plans for a phased reopening of schools in the fall of 2020; schools were fully opened by January of 2021 [32].

The primary objective of this analysis was to fill critical knowledge gaps by exploring key ASRH outcomes using nationally representative longitudinal data from Burkina Faso and Kenya, collected shortly before the COVID-19 pandemic and at two time points into the pandemic. We examined if ASRH outcomes and behaviors changed during the COVID-19 pandemic as predicted, including initiation of marriage/partnership, increased sexual activity, declines in modern contraception use, and increased pregnancies and unintended births. Our secondary objective was to assess if these outcomes varied by the unique contextual factors initiated by the COVID-19 pandemic, including (1) the household economic impact of the pandemic, (2) COVID-19 risk perception, and (3) COVID-19–related school disruptions.

## Methods

### Data

We used data from nationally representative panel surveys collected by the Performance Monitoring for Action (PMA) Project. PMA was initiated in 2013 with repeated cross-sectional surveys taking place on an annual cadence. Beginning in 2019, PMA changed to a longitudinal panel design with embedded annual cross-sectional surveys; the project has conducted three phases of longitudinal panel data collection to date. In Kenya and Burkina Faso, the first phase of panel data collection (herein referred to as “baseline”) occurred between November 2019 and February of 2020, prior to the global announcement of COVID-19 and the enactment of pandemic-related restrictions. The second phase of data collection took place approximately 1 year later between November 2020 and February 2021, and the third phase was completed during the same months between 2021 and early 2022.

Participants for PMA surveys were identified using a multistage stratified cluster design to draw a probability sample of households and women of childbearing age (15–49 years) in randomly selected enumeration areas. A cadre of trained female resident enumerators administered a household survey to selected households. The household survey is used to collect basic data on characteristics of all usual members of the household and identifies age-eligible women for the female survey. Consent for the household survey may be obtained verbally by any regular member of the household. Following completion of the household survey, enumerators approach eligible household women for administration of the female survey; consent is obtained orally and the survey is administered in a secure and private location in the household. Beginning in 2019, women who consented to a future PMA phase of data collection (follow-up) were automatically enrolled in the panel cohort and attempted contact at subsequent survey phases. More information about PMA is available in [33].

For this study, we used both PMA cross-sectional and panel data from 2016 to 2022. For the cross-sectional stage of this analysis (described further below), we included six rounds of repeated cross-sectional data from Kenya and Burkina Faso, from 2016 through 2021/2022, restricted to females aged 15–19 years (sample sizes range over time from 1,266 to 2,170 in Kenya and 729 to 1,490 in Burkina Faso). The longitudinal stage of this analysis involved the panel sample of adolescents from 2019 to 2021, restricted to adolescents aged 15–19 years at baseline (2019) who participated in at least one phase of follow-up. The baseline sample sizes for this research were 1,893 girls aged 15–19 years in Kenya and 1,422 girls aged 15–19 years in Burkina Faso.

Survey weights were generated in each geography adjusting for probability of selection and nonresponse at follow-up. More details on the PMA weighting procedures are available on the PMA website [34].

Ethical approval for the data collection activities, including informed consent procedures, was provided by three ethical review boards, including the Ethics Committee for Health Research at the Ministry of Health and Ministry of Higher Education, Scientific Research and Innovation in Burkina Faso; Kenyatta National Hospital-University of Nairobi Scientific Ethics Review Committee in Kenya; and Johns Hopkins Bloomberg School of Public Health.

### Measures

The primary measures of interest are a set of ASRH indicators used in family planning and adolescent health for national-level health monitoring and decision-making purposes [[35], [36], [37]]. This included (1) current partnership status (formal or informal partnership), (2) recent sexual activity, (3) modern contraception use, (4) currently pregnant or gave birth in the past year, and (5) unintended birth. Indicators were collected at all PMA phases, permitting us to examine change over time from before COVID-19 to after COVID-19 restrictions were lifted.

We assessed current partnership status by creating three measures using two survey items. Participants were asked if they were married or living with a partner as if married (formal partnership). Next, they were asked if they had a current boyfriend or partner (informal partnership). We created a dichotomous measure to assess formal partnerships, defined as marriage or living with a partner as if married, and a dichotomous measure to capture any partnership. Then, in the longitudinal sample, we measured initiation of partnership among participants who reported not being in a partnership at baseline, which we coded as 0 = remained single and 1 = initiated any form of partnership since 2019.

We investigated recent sexual activity by creating a binary measure capturing sex in the past four weeks. A secondary measure was created to assess recent sexual activity among sexually active adolescents who reported not being in formal unions (unmarried sexual activity). For this specific analysis, we only included the latter indicator given that premarital sexual debut is associated with relatively higher risk of unintended pregnancy, sexually transmitted diseases, and human immunodeficiency virus/acquired immune deficiency syndrome in some geographies in SSA [38,39]. We assessed current use of modern contraception at each phase by asking adolescents whether they or their partners were currently doing anything to delay or avoid getting pregnant, and, if so, what method they were using (following World Health Organization standards, modern methods in both settings included implants, intrauterine device, injectables, male/female condoms, pill, diaphragm, and/or emergency contraception). Less than 3% of women aged 15–24 years were using traditional contraceptive methods, so we instead focused on modern contraceptive use. Participants were asked if they were currently pregnant (yes/no) or had ever given birth (yes/no), and the date of their most recent birth. Given the small number of women in the sample who reported being currently pregnant, we created an aggregate “birth in the past year/currently pregnant” variable that captured both recent births and current pregnancies (1 = currently pregnant/birth in past 1 year, 0 = neither outcome). Then, currently pregnant women or women who reported ever having given birth were asked if their last birth/current pregnancy was wanted then, later, or not at all. Using this item, we created a dichotomous measure to capture birth intendedness, with 0 coded as intended (wanted then) and 1 coded as unintended (wanted later or not at all).

Next, we investigated whether COVID-19–related measures were associated with ASRH outcomes. The COVID-19 measures asked at Phase 2 included (1) household income loss due to COVID-19, (2) COVID-19 transmission concerns, and (3) school disruption. Household income loss was assessed through the question, “During the last 12 months, how much, if any, income loss did your household experience?” (none/partial/complete loss). Because perceived risk of infection is a precursor to behavior change [40], we included a measure of perceived COVID-19 risk, measured by asking, “How concerned are you about getting infected with Coronavirus (COVID-19) yourself?”; response categories included a three-point Likert scale ranging from “very concerned” to “not concerned at all”. Finally, a binary measure of school disruption was ascertained by asking girls who had reported attending school in the 12 months preceding the survey if they were attending school when COVID-19 restrictions were implemented (yes/no).

We included the sociodemographic control measures in regression models: age at baseline (15–19 years), urban/rural residence, highest level of education obtained (none, primary, secondary-inclusive of secondary one cycle and postprimary vocational, tertiary, or higher, inclusive of college or university), woman's parity (0, 1, 2+ children), and household wealth tertile (low, middle, high). Partnership status was included as a covariate in all models except those exploring it as the outcome of interest.

### Analytic approach

Analysis was conducted in three stages. First, to examine overall population-level changes in ASRH outcomes, we calculated the prevalence of primary outcome measures using repeated cross-sectional data from each of the six annual surveys conducted between 2016 and 2022. This provides basic descriptive patterns of changes from before the COVID-19 period through the COVID-19 period.

Second, using the longitudinal panel sample data, we pooled the three most recent phases of PMA data (2019, 2020, and 2021) and conducted random effects regression models, adjusting for respondent sociodemographic characteristics to account for changes in the composition of the PMA sample over time. The primary independent variable of interest was PMA survey year, which indicates whether there were statistically significant differences in outcomes between baseline (pre–COVID-19) and follow-up phases (2020 and 2021). Standard errors were adjusted to account for repeated measures for each woman aged 15–19 years. Given the low proportion of adolescents in the panel sample who reported a current or recent pregnancy/birth, birth intendedness was removed as a primary outcome of interest.

Finally, we assessed whether ASRH outcomes varied by the degree of COVID-19 impact using data from the second phase of longitudinal data collection (2020), where the COVID-19–related questions were included. We created four multivariable logistic regression models to examine the degree to which COVID-19 factors were associated with partnership initiation, recent sexual activity, modern contraception use, and pregnancy. Models were adjusted for baseline age, partnership status, household wealth index, educational attainment, and urban/rural residence. All analyses were conducted in Stata 16.1 [41].

## Results

Sociodemographic characteristics of the baseline panel sample are shown in Table 1. The mean age of participants was similar between Kenya and Burkina Faso, but we observed differences in educational attainment, with the majority of women aged 15–19 years having secondary education in Kenya but only primary or no education in Burkina Faso. Larger percentages of women aged 15–19 years were married and had children in Burkina Faso. The proportion of adolescents who reported their sexual debut was also higher in Burkina Faso than in Kenya (45.1% vs. 37.5%). About 75%–80% of women in the sample resided in rural areas in both contexts.

**Table 1:** Sociodemographic characteristics among adolescents aged 15–19 in Kenya and Burkina Faso, performance monitoring for action (PMA), 2019 (baseline)

	Kenya (n = 1,893)	Burkina Faso (n = 1,422)
Mean Age	16.9	17.0
Highest Level of Education		
None/Primary	38.0%	52.7%
Secondary	60.5%	47.0%
Tertiary	1.5%	0.4%
Relationship Status		
Single	63.6%	44.8%
Informal partnership/boyfriend	28.4%	27.6%
Married/living with partner	8.0%	27.6%
Residence		
Urban	21.1%	24.2%
Rural	78.9%	75.8%
Number of Children		
None	88.0%	83.2%
1	10.7%	14.2%
2+	1.4%	2.6%
Attended School in Past Year		
Yes	80.0%	64.5%
No	20.0%	35.5%
Household Wealth Tertile		
Low	41.3%	31.7%
Middle	36.7%	33.4%
High	22.1%	35.0%
Ever Sex		
Yes	37.5%	45.1%
No	62.5%	54.9%
Follow-Up (2020) COVID-19 Measures^a^		
Household Income Loss		
No household Income Loss	32.4%	63.5%
Partial household Income Loss	50.5%	30.1%
Total household Income Loss	17.1%	6.4%
COVID-19 Transmission Concerns		
Not Concerned/Little Concerned	6.0%	16.7%
Concerned	20.9%	16.6%
Very Concerned	73.1%	66.6%
School Disruption		
Yes – Experienced School Disruption	43.1%	41.7%

Figures may not add up to 100% due to rounding.

a COVID-19 measures are sourced from PMA Phase 2 Follow-Up; samples at Phase 2: Kenya n = 1,359; Burkina Faso n = 1,099.

### Repeated cross-sectional population-level ASRH trends (2016–2021)

Cross-sectional trends are presented in Table 2. The percentage of adolescents who were married or living with partner declined in both settings, dropping from 8.5% in 2016 to 4.6% in 2021 in Kenya and from 29.5% to 17.8% in Burkina Faso. There was a sustained increase in the proportion of contraceptive users in Burkina Faso over time that continued during the COVID years (from 9.8% in 2016 to 13.5% in 2022). In Kenya, the proportion of adolescents pregnant or who had given birth in the past year remained relatively stable from the pre-COVID year through the COVID years (from 8.0% in 2019 to 8.1% in 2022). In Burkina Faso, there was a marked decline in the proportion of adolescents who were currently pregnant or who had given birth in the past year between pre-COVID 2019 and COVID years (from 13.6% in 2019 to 8.2% in 2022), following a period of stability before 2019.

**Table 2:** Trends in female adolescent indicators (ages 15–19) in Kenya and Burkina Faso, PMA data 2016–21/22

Recent sexual activity is among women not in a formal union; unintended birth or pregnancy is among those who were currently pregnant or gave birth in the past year (sample sizes are listed in Table S1).

Data collected immediately before and during the COVID-19 pandemic are shaded.

PMA = Performance Monitoring for Action; SD = standard deviation.

### Longitudinal analysis of differences in ASRH outcomes before and during COVID-19 (2019–2021)

Longitudinal analysis (Table 3) revealed a statistically significant difference in partnership outcomes comparing pre–COVID-19 (2019) and COVID-19 time points (2020, 2021). There were fewer partnerships in 2021 as compared to pre-COVID in 2019 (Burkina Faso: adjusted odds ratio [aOR] 0.62; 95% confidence interval [CI]: 0.50–0.78; Kenya: aOR 0.64; 95% CI: 0.50–0.81) and significantly fewer formal partnerships in both 2020 and 2021 as compared to baseline. In Kenya, recent unmarried sexual activity was 43% higher in 2021 compared to 2019 (aOR 1.43; 95% CI: 1.03–1.98), but we also noted a significant increase in modern contraceptive (aOR 1.42; 95% CI: 1.03–1.97). In contrast, no changes in contraceptive use were noted in Burkina Faso. Finally, we found significant declines in recent birth or current pregnancy in both countries, with odds declining by 32% and 31% between 2019 and 2020 (COVID year), and 2021, in Kenya, and a 48% reduction between 2019 and 2021 in Burkina Faso.

**Table 3:** Differences in adolescent ASRH between before (2019) and during COVID-19 (2020, 2021), PMA Kenya and Burkina Faso

	Kenya		Burkina Faso	
	aOR	95% CI	aOR	95% CI
Any partnership (formal or informal)				
2019 (ref)	----	----	----	----
2020	1.13	0.94, 1.37	1.19	0.98, 1.45
2021	**0.62**	**0.50, 0.78**	**0.64**	**0.50, 0.81**
Formal Partnership				
2019 (ref)	----	----	----	----
2020	**0.43**	**0.28, 0.67**	**0.67**	**0.51, 0.89**
2021	**0.21**	**0.12, 0.36**	**0.35**	**0.25, 0.50**
Recent Unmarried Sexual Activity				
2019 (ref)	----	----	----	----
2020	0.93	0.69, 1.26	0.97	0.71, 1.32
2021	**1.43**	**1.03, 1.98**	1.15	0.80, 1.65
Modern Contraception				
2019 (ref)	----	----	----	----
2020	1.28	0.95, 1.72	1.06	0.82, 1.38
2021	**1.42**	**1.03, 1.97**	1.04	0.77, 1.42
Birth in the Past 1 Year				
2019 (ref)	----	----	----	----
2020	0.73	0.50, 1.05	0.93	0.66, 1.30
2021	0.83	0.55, 1.24	**0.57**	**0.37, 0.86**
Currently Pregnant				
2019 (ref)	----	----	----	----
2020	0.70	0.42, 1.16	**0.65**	**0.44, 0.97**
2021	**0.55**	**0.31, 0.99**	0.63	0.39, 1.01
Birth in the Past Year *or* Currently Pregnant				
2019 (ref)	----	----	----	----
2020	**0.68**	**0.50, 0.95**	0.76	0.57, 1.01
2021	**0.69**	**0.48, 0.99**	**0.52**	**0.36, 0.74**

Covariates include age, urban/rural, wealth tertile, education, and any partnership (not included in partnership models); parity is included only in the model for modern contraception use only; confidence intervals that do not cross 1.00 are shown in bold font.

aOR = adjusted odds ratio; CI = confidence interval; ASRH = adolescent sexual and reproductive health; PMA = Performance Monitoring for Action.

### Cross-sectional (2020) analysis of specific COVID-19 measures and SRH outcomes

The longitudinal analysis examining changes at the individual level are reported in Tables 4 and 5. While no significant change in premarital sexual activity was observed between 2019 and 2020, results show that unmarried Kenyan girls whose households experienced total household income loss were significantly less likely to have engaged in recent sexual activity (aOR = 0.47, 95% CI 0.25–0.87) as compared to girls who reported no household income loss. In terms of contraceptive use, Kenyan girls who experienced school disruption experienced declines in modern contraception in 2020 (aOR = 0.64, 95% CI 0.41–0.99), relative to those who did not. In Burkina Faso, school disruption was associated with higher likelihood of using modern contraception (aOR 2.25, 95% CI 1.10–4.60). Furthermore, Burkinabe girls who reported being very concerned about COVID-19 transmission had 2.19 times greater odds of using modern contraception as compared to girls who reported not being concerned (aOR = 2.19, 95% CI 1.12–4.28), but there was no such relationship in Kenya. Burkinabe girls who experienced total household income loss or school disruption were significantly less likely to have reported being currently pregnant in 2020 relative to those who had reported no household income loss or school disruption (aOR = 0.13, 95% CI 0.02–0.91 and aOR = 0.24, CI 0.07–0.91, respectively). We did not find a relationship between any of the COVID-19 factors and initiation of partnership in either Kenya or Burkina Faso between baseline and follow-up.

**Table 4:** Logistic regression results: differences in ASRH outcomes by COVID-19 impact, Kenya Phase 2 (2020) PMA survey

	Partnership	Recent unmarried sexual activity	Modern contraception use	Pregnant
	aOR (95% CI)	aOR (95% CI)	aOR (95% CI)	aOR (95% CI)
Baseline Age	**1.46 (1.30–1.65)**	**1.61 (1.37–1.85)**	**1.54 (1.36–1.75)**	1.08 (0.76–1.54)
Highest level of education				
None/Primary (ref)	----	----	----	----
Secondary	1.41 (0.87–2.27)	0.70 (0.43–1.15)	1.43 (0.94–2.17)	0.52 (0.23–1.19)
Tertiary or higher	1.65 (0.59–4.61)	1.25 (0.47–3.30)	1.38 (0.57–3.36)	0.72 (0.07–7.95)
Partnership Status				
Single (ref)	----	----	----	----
Married or partnered	----	----	1.34 (0.72–2.50)	60.47 (19.39–188.50)
Number of Children				
0 (ref)	----	----	----	----
1	----	**1.77 (1.01–3.13)**	**5.24 (3.29–8.34)**	**1.01 (0.03–0.31)**
2+	----	1.51 (0.23–10.06)	**6.31 (2.67–14.88)**	**0.03 (0.00–0.21)**
Residence				
Urban (ref)	----	----	----	----
Rural	1.36 (0.82–2.25)	0.92 (0.47–1.81)	0.80 (0.48–1.32)	0.85 (0.36–2.00)
Household Wealth Tertile				
Lowest (ref)	----	----	----	----
Middle	1.37 (0.93–2.01)	1.10 (0.71–1.70)	1.38 (0.85–2.25)	0.56 (0.24–1.32)
Highest	0.94 (0.55–1.61)	0.58 (0.34–1.00)	0.77 (0.45–1.32)	0.59 (0.20–1.77)
COVID-19 Factors				
Concerned about COVID Infection				
Not concerned (ref)	----	----	----	----
Concerned	0.90 (0.39–2.10)	**3.05 (1.18–7.91)**	0.92 (0.43–1.96)	**6.46 (1.22–34.27)**
Very concerned	0.93 (0.43–2.05)	2.45 (0.92–6.52)	1.31 (0.63–2.75)	3.08 (0.63–15.17)
Household COVID-19 economic impact				
No household income loss (ref)	----	----	----	----
Partial household income loss	0.91 (0.58–1.43)	0.99 (0.61–1.62)	1.03 (0.64–1.67)	1.13 (0.50–2.52)
Total household income loss	0.74 (0.43–1.25)	**0.47 (0.25–0.87)**	0.73 (0.37–1.43)	0.69 (0.20–2.34)
School disruption				
No school disruption (ref)	----	----	----	----
School disruption	1.05 (0.72–1.51)	0.84 (0.52–1.35)	**0.64 (0.41–0.99)**	1.32 (0.57–3.01)
n = 1,359	n = 885	n = 1,219	n = 1,343	n = 1,354

All analyses were weighted for study design and nonresponses; “partnership” is measured among women who were single at baseline in 2019; confidence intervals that do not cross 1.00 are shown in bold font.

aOR = adjusted odds ratio; ASRH = adolescent sexual and reproductive health; CI = confidence interval; PMA = Performance Monitoring for Action.

**Table 5:** Logistic regression results: differences in ASRH outcomes by COVID-19 impact, Burkina Faso Phase 2 (2020) PMA survey

	Partnership	Recent unmarried sexual activity	Modern contraception use	Pregnant
	aOR (95% CI)	aOR (95% CI)	aOR (95% CI)	aOR (95% CI)
Baseline Age	**1.24 (0.98–1.57)**	**1.46 (1.20–1.77)**	**1.47 (1.23–1.74)**	0.86 (0.67–1.09)
Highest level of education				
None/Primary (ref)	----	----	----	----
Secondary	1.32 (0.66–2.71)	1.48 (0.73–2.97)	1.15 (0.72–1.85)	2.00 (0.89–4.50)
Tertiary or higher	1.03 (0.21–5.07)	2.02 (0.55–7.48)	1.20 (0.44–3.23)	----
Partnership Status				
(ref) Single	----	----	----	----
Married or partnered	----	----	0.98 (0.33–2.91)	**45.64 (10.51–198.18)**
Number of Children				
0 (ref)	----	----	----	----
1	----	0.81 (0.28–2.30)	**2.54 (1.07–6.02)**	**0.12 (0.06–0.22)**
2+	----	1.12 (0.04–28.5)	**5.55 (1.47–21.00)**	**0.12 (0.12–0.87)**
Residence				
Urban (ref)	----	----	----	----
Rural	0.67 (0.29–1.59)	1.02 (0.57–2.52)	0.77 (0.40–1.49)	1.47 (0.56–3.85)
Household Wealth Tertile				
Lowest (ref)	----	----	----	----
Middle	1.01 (0.42–2.46)	1.20 (0.57–2.52)	0.92 (0.47–1.80)	0.89 (0.34–2.33)
Highest	0.71 (0.26–1.96)	0.54 (0.24–1.25)	0.81 (0.36–1.84)	1.27 (0.37–4.39)
COVID-19 Factors				
Concerned about COVID Infection				
Not concerned (ref)	----	----	----	----
Concerned	1.21 (0.50–2.94)	0.97 (0.38–2.48)	1.60 (0.65–3.94)	3.06 (0.77–12.14)
Very concerned	1.19 (0.62–2.29)	1.12 (0.50–2.50)	**2.19 (1.12–4.28)**	2.72 (0.77–9.64)
Household COVID-19 economic impact				
No household income loss (ref)	----	----	----	----
Partial household income loss	1.25 (0.68–2.30)	1.50 (0.77–2.93)	1.04 (0.62–1.75)	0.93 (0.36–2.38)
Total household income loss	2.06 (0.87–4.90)	1.09 (0.21–5.58)	1.37 (0.64–2.92)	**0.13 (0.02–0.91)**
School disruption				
No school disruption (ref)	----	----	----	----
School disruption	0.75 (0.43–1.33)	0.59 (0.05–1.13)	**2.25 (1.10–4.60)**	**0.24 (0.07–0.91)**
n = 1,099	n = 468	n = 800	n = 1,046	n = 1,057

All analyses were weighted for study design and nonresponses; “partnership” is measured among women who were single at baseline in 2019; confidence intervals that do not cross 1.00 are shown in bold font.

aOR = adjusted odds ratio; CI = confidence interval; ASRH = adolescent sexual and reproductive health; PMA = Performance Monitoring for Action.

## Discussion

The goal of this research was to measure changes in ASRH outcomes in Kenya and Burkina Faso from before and 1–2 years into the global COVID-19 pandemic period by leveraging the power of longitudinal panel data. At the onset of the coronavirus pandemic, many feared that COVID-19 would have a profound influence on the health and well-being of adolescents in SSA [1,4], but there are few data sources available to explore changes in these outcomes during this period. We used multiple phases of nationally representative data, including both repeated cross-sectional and longitudinal panel data, to address this research gap.

Overall, we found very limited evidence of an impact of the COVID-19 period on ASRH trends among adolescents in these settings. The few significant changes were, in fact, in the opposite direction than what was predicted: partnerships (formal and informal) were less common across both settings in 2021 compared to pre–COVID-19 2019, modern contraceptive use increased in Kenya, and adolescent pregnancies and births were less common in both 2020 and 2021 in Kenya, and in 2021 in Burkina Faso. These results are also internally consistent: with fewer partnerships and increased contraceptive use, one would expect fewer pregnancies and births.

When we explore how these outcomes varied by degree of COVID-19 impact, findings are mixed, and show that COVID-19 did not have the ubiquitous effect that many predicted. While Kenyan adolescents who experienced school disruption were less likely to be using modern contraception, adolescents in Burkina Faso who experienced school disruption had a higher likelihood of using a modern contraception and a lower likelihood of being currently pregnant. Perhaps surprisingly, we did not find a relationship between school disruption and initiation of partnership or recent sexual activity in either setting, which counters research on the relationship between school disruptions and early marriage/pregnancy as well as past projections on the relationship between the pandemic and early marriage [7]. Furthermore, in Kenya, we see that unmarried adolescents whose households experienced total income loss were in fact less likely to have engaged in recent sexual activity; in Burkina Faso, we see a similar relationship between total household income loss and lowered odds of pregnancy.

Although these findings are contrary to initial expectations of the pandemic's negative impact on adolescent health, they are consistent with the global body of emerging research on the mixed relationship between COVID-19 and family planning usage. Studies of adult women aged 15–49 years in the region showed no declines in contraceptive use due to COVID-19 [19,20,42] as we find here.

We suspect that the stability in contraceptive use can be explained by a few reasons. First, studies of contraceptive services revealed that the supply of contraceptives at healthcare facilities recovered after short-lived stockouts early in the pandemic, hence access to contraceptives was not a long-lasting problem in many geographies [17,43]. Furthermore, given stigma related to premarital adolescent sexual activity, many adolescents may access family planning services through means other than the formal health sector, such as obtaining condoms from shops or other private sector options, thus reducing the impact of formal service disruptions on pregnancy and contraceptive behaviors within this population. Research has speculated that women in SSA are conditioned to expect intermittent shortages of contraceptive methods and adjust their behavior accordingly by finding alternative solutions [42,44]. Finally, in both countries, there have been recent gains in the health landscape, including universal access to contraception implemented in Burkina Faso in 2020 and a resumption of service delivery after significant social movements in healthcare facilities throughout Kenya [45,46]; the rapidly evolving healthcare climates, and social movements, may have been able to absorb the COVID-19 shockwaves and protect against the negative outcomes of COVID-19 more so than initially predicted or expected.

Although this study benefits from the use of longitudinal panel data for adolescents before and during COVID-19, and nationally representative data that are broader in scope than many other studies, there are some limitations in this research. First, we do not use statistical methods that allow us to establish a causal relationship between COVID-19 and ASRH outcomes. In addition, the number of pregnancies or births was too small for us to conduct regression analysis of unintended births or pregnancies in these regions. For this research, we rely on retrospective self-reports of ASRH outcomes, and such measures may not be completely accurate. Finally, there are indeed likely other ways in which COVID-19 impacted SRH outcomes, but we only focus on three key COVID-19 mechanisms: school disruptions, economic impact, and concern about COVID-19 infection.

Using data from PMA, we did not find evidence of a substantial impact of COVID-19 on some ASRH outcomes in Kenya and Burkina Faso in the relatively early stages of the pandemic; our findings suggest that the pandemic did not significantly shift the trajectory of ASRH trends or lead to devastating reversals of public health progress after pandemic restrictions were lifted. As such, youth in Burkina Faso and Kenya appear to have avoided a detrimental effect of COVID-19 on some ASRH outcomes such as early pregnancy and initiation of early formal partnership; much of the concern regarding widespread and sustained negative impact appears to be largely misguided. While we did not identify a significant impact of the pandemic on some ASRH outcomes in Kenya and Burkina Faso in the relatively early stages of COVID-19, this does not mean that other family planning, health, or social behaviors and outcomes were not affected. In fact, as described above, the impact of COVID-19 was nuanced and inconsistent, influencing some measures in certain geographics but not others. Given that the focus of this research does not extend past early 2022, understanding the pandemic-related health impacts on ASRH behaviors and long-term trajectories remains a key area for continued investigation. As such, adolescent populations warrant continued monitoring by the global health community to ensure that ASRH goals are equitably attained.

## Availability of Data and Materials

The datasets supporting the conclusions shared in this article are available upon request.

## References

[1] Addae E.A. (2021). COVID-19 pandemic and adolescent health and well-being in sub-Saharan Africa: Who cares?. Int J Health Plann Manage.

[2] Cluver L., Lachman J.M., Sherr L. (2020). Parenting in a time of COVID-19 [published correction appears in Lancet. 2020 Apr 11;395(10231):1194]. Lancet.

[3] Hussein J. (2020). COVID-19: What implications for sexual and reproductive health and rights globally?. Sex Reprod Health Matters.

[4] Oppong Asante K., Quarshie E.N., Andoh-Arthur J. (2021). COVID-19 school closure and adolescent mental health in sub-Saharan Africa. Int J Soc Psychiatry.

[5] Partridge-Hicks S. (2020). Rise in teenage pregnancies in Kenya linked to COVID-19 lockdown. Global Citizen. https://www.globalcitizen.org/en/content/rise-in-teenage-pregnancies-during-kenya-lockdown/.

[6] Riley T., Sully E., Ahmed Z., Biddlecom A. (2020). Estimates of the potential impact of the COVID-19 pandemic on sexual and reproductive health in low- and middle-income countries. Int Perspect Sex Reprod Health.

[7] Murewanhema G. (2020). Adolescent girls, a forgotten population in resource-limited settings in the COVID-19 pandemic: Implications for sexual and reproductive health outcomes. Pan Afr Med J.

[8] Yukich J., Worges M., Gage A.J. (2021). Projecting the impact of the COVID-19 pandemic on child marriage. J Adolesc Health.

[9] Lindberg L.D., Bell D.L., Kantor L.M. (2020). The sexual and reproductive health of adolescents and young adults during the COVID-19 pandemic. Perspect Sex Reprod Health.

[10] Meherali S., Adewale B., Ali S. (2021). Impact of the COVID-19 pandemic on adolescents’ sexual and reproductive health in low- and middle-income countries. Int J Environ Res Public Health.

[11] Austrian K., Pinchoff J., Tidwell J.B. (2020). COVID-19 related knowledge, attitudes, practices and needs of households in informal settlements in Nairobi, Kenya. Lancet.

[12] Decker M.R., Wood S.N., Thiongo M. (2021). Gendered health, economic, social and safety impact of COVID-19 on adolescents and young adults in Nairobi, Kenya. PLoS One.

[13] Gittings L., Toska E., Medley S. (2021). 'Now my life is stuck!': Experiences of adolescents and young people during COVID-19 lockdown in South Africa. Glob Public Health.

[14] Khan S., Kemigisha E., Turyakira E. (2022). Dramatic effects of COVID-19 public health measures and mass reverse migration on youth sexual and reproductive health in rural Uganda. Paediatr Child Health.

[15] Karp C., Moreau C., Sheehy G. (2021). Youth relationships in the era of COVID-19: A mixed-methods study among adolescent girls and young women in Kenya. J Adolesc Health.

[16] Zulaika G., Bulbarelli M., Nyothach E. (2022). Impact of COVID-19 lockdowns on adolescent pregnancy and school dropout among secondary schoolgirls in Kenya. BMJ Glob Health.

[17] Fuseini K., Jarvis L., Ankomah A. (2022). Did COVID-19 impact contraceptive uptake? Evidence from Senegal. Stud Fam Plann.

[18] Makumbi F., Sebina Kibira S.P., Giibwa L. (2021).

[19] Karp C., Wood S.N., Guiella G. (2021). Contraceptive dynamics during COVID-19 in sub-Saharan Africa: Longitudinal evidence from Burkina Faso and Kenya. BMJ Sex Reprod Health.

[20] Wood S.N., Karp C., OlaOlorun F. (2021). Need for and use of contraception by women before and during COVID-19 in four sub-Saharan African geographies: Results from population-based national or regional cohort surveys. Lancet Glob Health.

[21] Mbushi F., Wyss N., Cho E. (2022).

[22] UNICEF (2020). https://www.unicef.org/reports/country-regional-divisional-annual-reports-2020/Burkina-Faso.

[23] INSD et ICF (2022).

[24] Kenya National Bureau of Statistics, Ministry of Health of Kenya, National AIDS Control Council/Kenya (2015).

[25] Kenya National Bureau of Statistics and ICF (2023).

[26] Institut Supérieur des Sciences de la Population (ISSP), Université Joseph Ki-Zerbo, Ouagadougou, Burkina Faso, The Bill & Melinda Gates Institute for Population and Reproductive Health at the Johns Hopkins Bloomberg School of Public Health, Jhpiego (2022).

[27] International Centre for Reproductive Health Kenya (ICRHK), the Bill & Melinda Gates Institute for Population and Reproductive Health at the Johns Hopkins Bloomberg School of Public Health, Jhpiego (2022).

[28] Zidouemba P.R., Kinda S.R., Ouedraogo I.M. (2020). Could Covid-19 worsen food insecurity in Burkina Faso?. Eur J Dev Res.

[29] Sory O., Kiendrébéogo J.A., Kafando Y. (2023). The role and contribution of civil society and community actors in COVID-19 prevention and control: The case of the COMVID COVID-19 movement in Burkina Faso. BMJ Glob Health.

[30] UNICEF (2020). https://www.unicef.org/documents/burkina-faso-humanitarian-situation-report-june-2020.

[31] ACAPS (2020). COVID-19: Insecurity and education in Burkina Faso – situation at the start of the new school year. http://www.acaps.org/fileadmin/Data_Product/Main_media/20201015_acaps_thematic_series_on_education_burkina_faso.pdf.

[32] Nudungu T. (2020). Schools to re-open officially on January 4- CS Magoha. Citizen TV. https://www.citizen.digital/news/schools-to-re-open-officially-on-january-4-cs-magoha-519557.

[33] Zimmerman L., Olson H., PMA2020 Principal Investigators Group (2017). PMA2020: Rapid turn-around survey data to monitor family planning service and practice in ten countries. Stud Fam Plann.

[34] (2023). PMA data. Bill & Melinda Gates Institute for Population and Reproductive Health at the Johns Hopkins Bloomberg School of Public Health and Jhpiego. https://www.pmadata.org/data.

[35] (2023). FP2030 – What we measure. FP2030. https://fp2030.org/what-we-measure.

[36] Newby H., Marsh A.D., Moller A.B. (2021). A scoping review of adolescent health indicators. J Adolesc Health.

[37] World Health Organization (2020). https://www.who.int/docs/default-source/mca-documents/advisory-groups/gama/gama-list-of-indicators-draft-2-v20201020.pdf?sfvrsn=f6d00176_6.

[38] Bain L.E., Zweekhorst M.B.M., de Cock Buning T. (2020). Prevalence and determinants of unintended pregnancy in Sub-Saharan Africa: A systematic review. Afr J Reprod Health.

[39] Ikamari L., Izugbara C., Ochako R. (2013). Prevalence and determinants of unintended pregnancy among women in Nairobi, Kenya. BMC Pregnancy Childbirth.

[40] Rosenstock I.M., Strecher V.J., Becker M.H. (1988). Social learning theory and the health belief model. Health Educ Q.

[41] StataCorp (2020).

[42] Moreau C., Karp C., Wood S. (2023). Trends in fertility intentions and contraceptive practices in the context of COVID-19 in sub-Saharan Africa: Insights from four national and regional population-based cohorts. BMJ Open.

[43] Leight J., Hensly C., Chissano M., Ali L. (2021). Short-term effects of the COVID-19 state of emergency on contraceptive access and utilization in Mozambique. PLoS One.

[44] Assefa N., Sié A., Wang D. (2021). Reported barriers to healthcare access and service disruptions caused by COVID-19 in Burkina Faso, Ethiopia, and Nigeria: A telephone survey. Am J Trop Med Hyg.

[45] Browne L., Cooper S., Tiendrebeogo C. (2022). Using experience to create evidence: A mixed methods process evaluation of the new free family planning policy in Burkina Faso. Reprod Health.

[46] Scanlon M.L., Maldonado L.Y., Ikemeri J.E. (2021). A retrospective study of the impact of health worker strikes on maternal and child health care utilization in Western Kenya. BMC Health Serv Res.

