# Supplementary material for: Trends in Adolescent Sexual and Reproductive Health Outcomes Before and Into the COVID-19 Pandemic in Burkina Faso and Kenya: Evidence From Panel Data
Source: J Adolesc Health. 2024 Aug;75(2):344–52. doi: 10.1016/j.jadohealth.2024.04.023 (PMC11252117; doi:10.1016/j.jadohealth.2024.04.023)
Supplement: Table S1 [file mmc1.docx]

**Supplemental Table: Sample Sizes for restricted variable – Recent Sexual Activity and Unintended birth/pregnancy.**

| **Kenya** | **PMA Round 5 November – December 2016** | **PMA Round 6 November – December 2017** | **PMA Round 7 November – December 2018** | **PMA Phase 1 November – December 2019** | **PMA Phase 2 November – December 2020** | **PMA Phase 3 November – December 2021** |
| --- | --- | --- | --- | --- | --- | --- |
| Recent unmarried sexual activity n = | 1178 | 1169 | 1176 | 1906 | 2028 | 1995 |
| Unintended birth or pregnancy n = | 117 | 97 | 118 | 165 | 172 | 182 |
| **Burkina Faso** | **PMA Round 4 November 2016 – January 2017** | **PMA Round 5 November 2017 – January 2018** | **PMA Round 6 December 2018 – January 2019** | **PMA Phase 1 December 2019 – February 2020** | **PMA Phase 2 December 2020 – February 2021** | **PMA Phase 3 December 2021 – February 2022** |
| Recent unmarried sexual activity n = | 548 | 618 | 599 | 1186 | 1161 | 1136 |
| Unintended birth or pregnancy n = | 102 | 111 | 107 | 173 | 149 | 124 |
